# Seagrasses provide a novel ecosystem service by trapping marine plastics

**DOI:** 10.1038/s41598-020-79370-3

**Published:** 2021-01-14

**Authors:** Anna Sanchez-Vidal, Miquel Canals, William P. de Haan, Javier Romero, Marta Veny

**Affiliations:** 1 GRC Geociències Marines, Departament de Dinàmica de la Terra i de l’Oceà, Universitat de Barcelona, 08028 Barcelona, Spain; 2 Departament de Biologia Evolutiva, Ecologia i Ciències Ambientals, Universitat de Barcelona, 08028 Barcelona, Spain

**Keywords:** Environmental impact, Ocean sciences

## Abstract

There is strong evidence that the seafloor constitutes a final sink for plastics from land sources. There is also evidence that part of the plastics lying on the shallow seafloor are washed up back to the shoreline. However, little is known on the natural trapping processes leading to such landwards return. Here we investigate microplastics and larger plastic debris within beached seagrass remains including balls (aegagropilae) made of natural aggregates of vegetal fibers intertwined by seawater motion. We found up to 1470 plastic items per kg of plant material, which were mainly composed of negatively buoyant polymer filaments and fibers. Our findings show that seagrass meadows promote plastic debris trapping and aggregation with natural lignocellulosic fibers, which are then ejected and escape the coastal ocean. Our results show how seagrasses, one of the key ecosystems on Earth in terms of provision of goods and services, also counteract marine plastic pollution. In view of our findings, the regression of seagrass meadows in some marine regions acquires a new dimension.

## Introduction

Microplastics -plastic particles smaller than 5 mm in size^1^-derive from fragmentation and degradation of large plastic items^2,3^, and also from direct manufacturing of microscopic particles such as virgin plastic pellets, cosmetic microbeads and clothing microfibres^4,5^. Research on microplastic pollution has long focused on sea surface accumulations^6–8^. However, there is a growing body of evidence that floating plastic debris account for less than 1% of the global ocean plastic inventory^9^, whereas the vast majority sinks to the seafloor^1,10,11^. Microplastics have indeed been found in all marine environments, shallow and deep, close to shore and amidst ocean basins^11–15^. Further, recent studies have shown that bottom currents control the distribution of microplastics on the seafloor, transporting them from shallow to deep waters where they accumulate^14,16^. In this study, we provide evidence of the entrapment of plastic debris from the shallow marine environment by seagrasses. This represents a continuous purge of plastic debris out of the sea that has been omitted in surface (nearshore to offshore) and bottom (shallow to deep) simulations of microplastics transport^3,8,17,18^.

Seagrass meadows are widespread in shallow coastal waters^19^ and provide important ecosystem services and benefits, such as water quality improvement^20^, CO_2_ absorption^21^, climate change mitigation^22^, sediment production for seafloor and beach stabilization^23^, coastal protection^24^, nursery and refuge areas for many species^25^, and support in fisheries production^26^. We have investigated microplastics and larger plastic debris washed ashore together with natural debris of the seagrass *Posidonia oceanica*, a Mediterranean endemic seagrass forming lush, extensive meadows from 0.5 to 40 m of water depth. According to the latest and more accurate estimate the total area covered by *P. oceanica* is 1.2 M Ha^27^. *P. oceanica* has long, ribbon-like leaves, with a clear differentiation in leaf blade (photosynthetic) and leaf base or leaf sheath (non pigmented and fibrous) that attaches the leaf to the stem, called rhizome^28^.

As a temperate species, *P. oceanica* loses leaves in autumn, which are washed by waves and currents and accumulate on adjacent beaches as wrack beds. These vegetal deposits, besides attenuating wave energy, protecting the shoreline and preventing coastal erosion, influence also dune vegetation not only by providing it with nutrients but also by preventing substrate aridity^29^. In addition, in this species (as in other congenerics in the southern hemisphere) leaf sheaths remain attached to the rhizome when leaves shed, and are slowly buried by sedimentation in the so called “matte”, an accumulation of dead rhizomes and roots that can persist for millennia^30^. During the burial process, leaf sheaths, which are rich in lignocellulose, suffer mechanical erosion, releasing part of the constituent fibers that intertwine to form ball-shaped agglomerates known as seaballs, Neptune balls or aegagropilae (EG)^31^. These balls are also washed ashore. While leaf sheaths are present in almost all seagrass genera, only sheath cells in *Posidonia* have thin and lignified walls^28^, and thus fibers provide the needed stiffness to form EG^32^. The genus *Posidonia* has a unique fragmented distribution in the temperate waters of the Mediterranean Sea and southern Australia. Regrettably, it is estimated that between 13 and 50% of potential initial *P. oceanica* area may have been lost since 1960^27,33^.

To examine the role played by these piles of vegetal remains in trapping and extracting plastic debris from sea and carrying them to shore we have examined both EG and beach wracks accumulated on different beaches of Mallorca Island, in the Western Mediterranean Sea. This island, 3640 km^2^ in area and ca. 560 km in perimeter, is an optimal site to address these issues, because of both the extensive meadows of *P. oceanica* in its waters^34^ and the fact that the highest accumulations of floating plastic debris in the Mediterranean Sea occur in its nearshore^35^.

## Results and discussion

Plastic debris in loose leaves (wracks) were found in 50% of the samples, with up to 613 plastic items per kg of dead leaves. Plastic items consisted mostly of fragments (61.29%) followed by pellets (33.67%) and foams (2.90%). The polymers were identified by spectrometry and included polyethylene (PE) (50.57%) followed by polypropylene (PP) (32.18%) and polyvinyl chloride (PVC) (6.90%). Plastic sizes ranged from 0.55 to 287 mm and averaged 9.08 mm.

**Figure 1:**
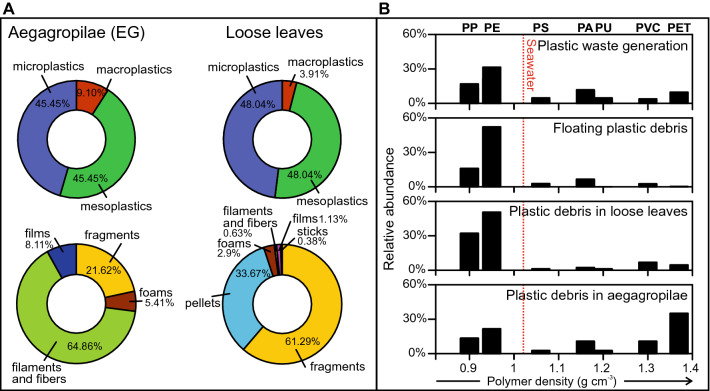
Plastic debris in seagrass remains. (**A**) Pie chart illustrating size of plastic debris (microplastics < 5 mm, mesoplastics 5–25 mm, and macroplastics > 25 mm), and type of plastic (filaments and fibers, fragments, films, foams, pellets and sticks) found in beached aegagropilae (EG) and loose leaves (wracks). (**B**) Relative abundance of each plastic polymer in plastic waste generation, floating plastic debris, plastic debris in loose leaves and aegagropilae (EG) vs. polymer density. Plastic waste generation is from Geyer et al.^64^, and plastic polymers floating at the sea surface are from Suaria et al.^43^. Only plastic polymers with percentages > 1% in at least two matrices (floating, leaves, EG) are taken into account. *PA* polyamide, *PE* polyethylene, *PET* polyethylene terephthalate, *PP* polypropylene, *PS* polyestyrene, *PU* polyurethane, *PVC* polyvinyl chloride.

Plastics of different sizes were found intertwined in 17% of the inspected EG (Fig. 1A, Table S2), with up to 1,470 plastic items per kg of dead seagrass remains. The plastic debris found were mostly filaments and fibers (64.86%), fragments (21.62%), films (8.11%) and foams (5.41%) (Fig. 1A). The polymers identified by spectrometry (n = 124) included polyethylene terephthalate (PET) (35.14%), PE (21.62%), PP (13.51%), polyamide (PA) (10.81%) and PVC (10.81%) (Fig. 1B). Plastic sizes ranged from 1.05 to 59.02 mm and averaged 9.48 mm.

Plastic debris weight represented < 1.6% and < 0.15% of the mass of dead leaves and EG, respectively. Plastic weight ranged 0–13.3 mg per EG unit.

**Figure 2:**
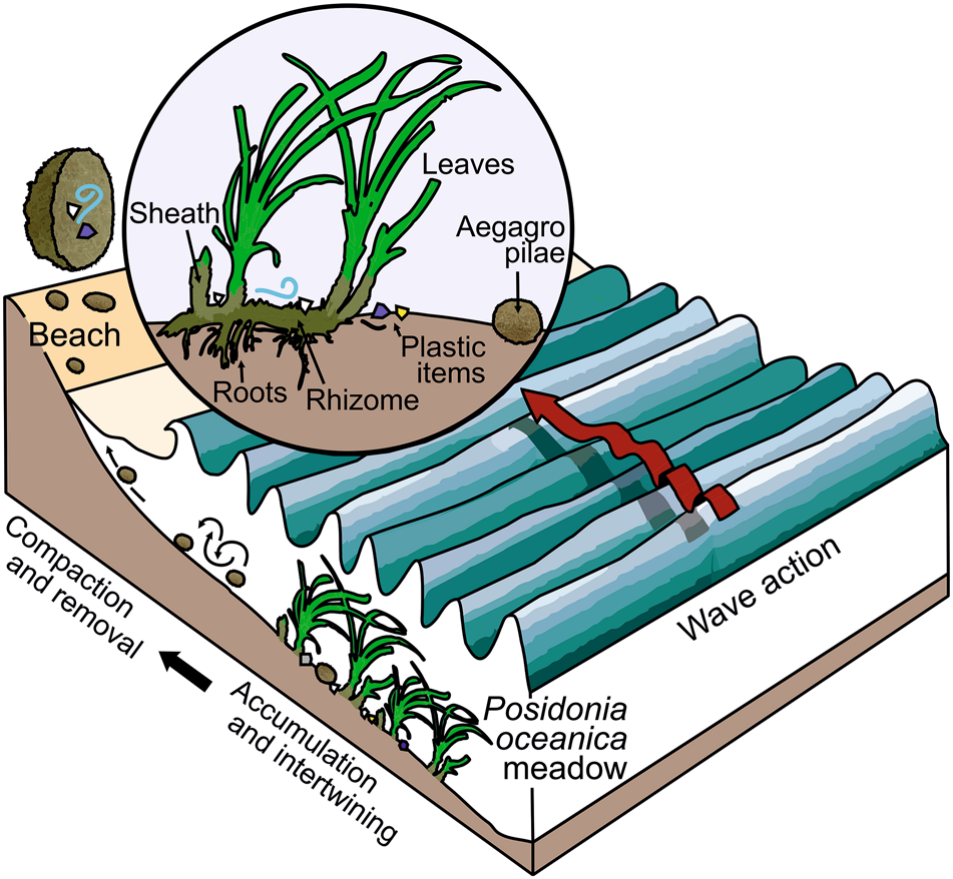
Trapping of plastic debris by seagrasses. Representation of the processes involved in the accumulation and intertwining of plastic items and sheath fibers to form plastic-rich aegagropilae (EG) found stranded in beaches.

Our findings demonstrate that high density plastic items in shallow meadows are intertwined, and subsequently trapped, with the lignocellulosic debris of the seagrass *P. oceanica* to form EG, which are washed ashore by sea waves mainly during stormy conditions (Fig. 2). Indeed, seagrass beds are known to promote deposition and reduce resuspension of sedimentary particles as a result of the reduction of water flow, turbulence and wave action by the plant canopies^36^. In addition, particle collision with dense seagrass canopies plays a role in sediment transport to the seabed and extraction from the water column^37^. Sediment trapping by seagrass meadows may also include plastic debris. Huang et al.^38^ found that microplastics in seagrass meadows were enriched by a factor up to 2.9 compared to non-vegetated areas. The reduction of the flow and the trapping effect of canopies may create soft bottom accumulations of plastic debris, deposited and stranded on the seabed, from where they can hardly escape. Accordingly, a statistically significant higher loading of polymers denser than seawater such as PET, PA, and PVC (Chi-square test = 22.6, df = 1, p < 0.001) were found in EG (Fig. 1B). Their negative buoyancy may have favoured their accumulation and aggregation with natural fibres in the meadow. Low density PE and PPs, which float in sea water, still represented a significant proportion among EG trapped plastic debris. Positively buoyant plastic debris may have reached the seabed due to collision with seagrass leaves, momentum loss and particle deposition^37^, or due to buoyancy loss because of biofouling^39^ or aggregation^40^.

**Figure 3:**
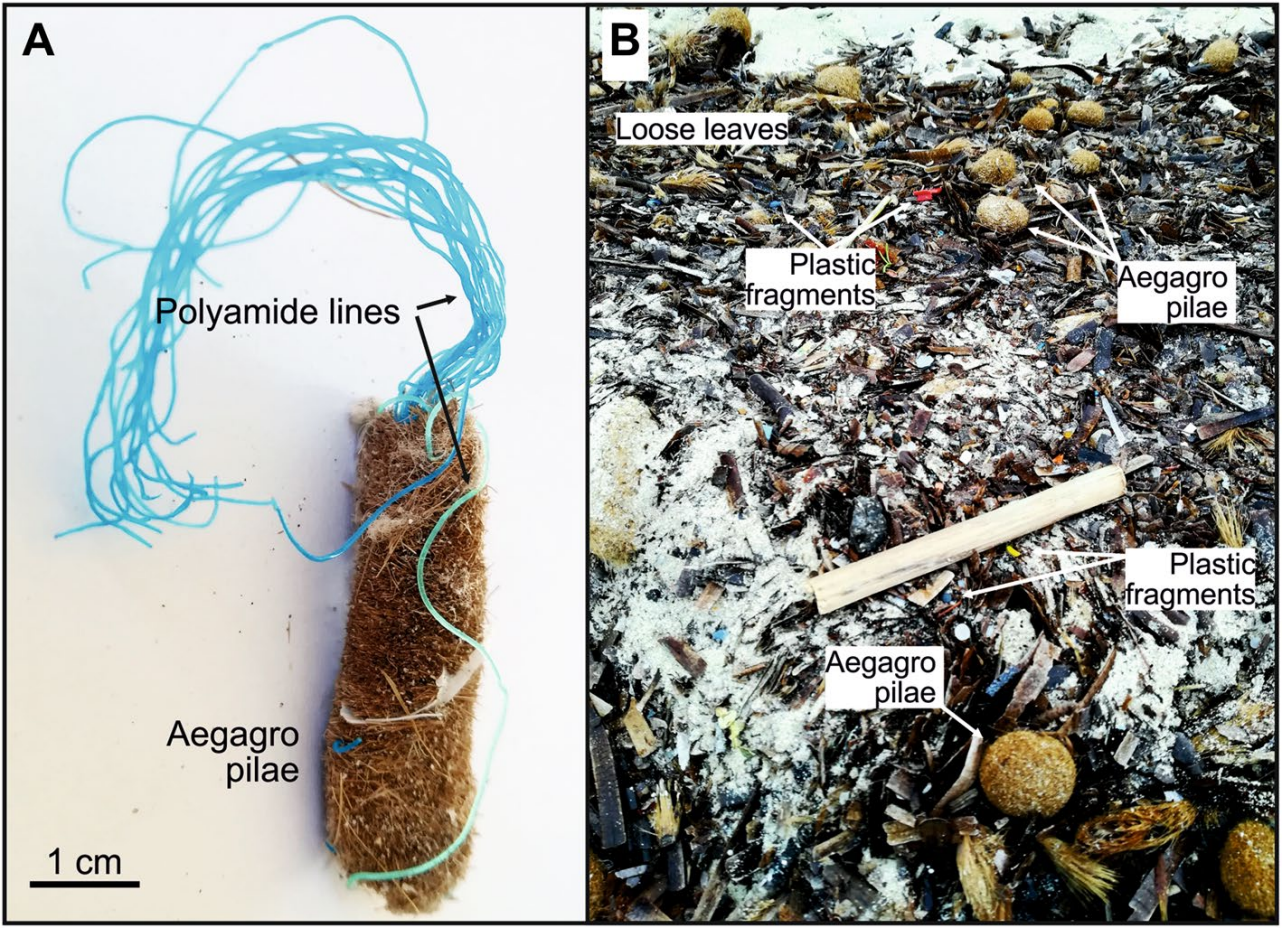
Plastic debris in aegagropilae (EG) and loose leaves found in beaches. (**A**) Polyamide filaments trapped in aegagropilae (EG), and (**B**) beached EG and loose leaves along with plastic debris.

In the seabed, it is likely that agitation caused by sea motion aggregates and holds together sheath fibers and plastic debris forming EG (Fig. 2). It has been suggested that EG formation is initiated at low abundances of vegetal fibers^41^, and that they grow by random aggregation of smooth fibers under relatively calmed conditions and for a long period of time^32^. Then, on a shorter time, sudden change in sea motion may cause repeated collisions of the EG with the seabed subsequently inducing the formation of an outward dense shell (*hardening*), which in turn, inhibits addition of new material^32^. EG size was observed to be inversely correlated with plastic abundance (r(27) = − 0.30, p < 0.01; including only EG with plastic presence). This suggests that the relatively rigidity of plastic debris, and specially polyamide filaments from fishing lines (Fig. 3), may decrease the natural tendency of the lignocellulosic fibers to agglomerate to form balls stiff when agitated by sea motion.

Plastic debris abundance per mass unit in loose dead leaves was significantly lower than those in EG (p < 0.001, Mann–Whitney U test) and mostly composed of low-density polymers (PE and PP) fragments and pellets (Fig. 1, Table S1). This suggests that plastic debris found stranded in wracks are, for the most, those drifting at the sea surface, which are transported close to shore by surface currents and wind waves, and are washed ashore at the same time as leaf litter. Indeed, similar low density polymer contributions have been found floating off the investigated beaches^35^ and drifting in open waters of the Mediterranean Sea^43^ (Fig. 1B). The occurrence of an extraordinary convective rainfall event in Mallorca Island in autumn 2017^44^ may had loaded coastal waters with an extra amount of PP pellets (Supplementary Table 2). Drifting floating plastic items were flushed ashore during autumn storms, alongside with seagrass remains from natural massive seasonal leaf loss^45^. Interestingly, plastic debris in wracks along the shoreline undergo photo-oxidative degradation and gradual fragmentation^46^, and may be eventually backwashed to the coastal sea and transported seaward by swash waves^47^. Accordingly, low-density plastic debris found in wracks show smaller size (Fig. 1) and thus higher deterioration than those in EG. In contrast, enclosure of plastic debris in EG protects them from exposure to UV radiation and mechanical abrasion and thus from breakdown into smaller sized pieces.

The fate of the EG is an open question. On the one hand, and in the context of beach residues management, one possibility would be to remove the balls to eliminate their associated plastic debris. However, it would be difficult to remove the balls without removing the stranded leaf litter, which is known to protect beaches against erosion^48^, provide nutrients for dune plants^29^ and feed beach arthropod communities^49^. On the other hand, the dense outward shell of the EG^32,42^, the refractory character of their lignocellulosic fibers^50^ and their low N content^51^ provide them with a high stiffness and resistance to degradation. Thus, the probability of a plastic debris being disentangled and degraded and/or backwashed may be relatively low. In any case, what happens to the plastic debris in the EG once ashore deserves further investigation.

Our understanding of plastic fluxes and pathways is incomplete. There appears to be a considerable proportion of all plastic dumped into the ocean that is missing as it has not been found in surveys tracking floating plastic debris^3,6–9^. Where this “missing” plastic is has been a longstanding scientific question after being posed by Thompson et al.^1^. During more than 15 years, an increasing number of studies have supported the idea that most plastic ends up in the seafloor^11,13,15^, acting as the ultimate reservoir of our throw-away society. However, other processes may also account for some of this discrepancy between estimations of plastics dumped and plastics floating at the sea surface. There are evidences that a natural sorting for plastic debris is occurring in coastal environments, where a major part of entering plastics are stranded and captured, and only a small fraction escapes offshore^52^. Here we show that plastic debris in the shallow seafloor could be trapped in seagrass remains, eventually leaving the marine environment through beaching. Furthermore, taking an average plastic debris abundance in EG of 57.8 items kg^−1^, and a Mediterranean basin sheath fiber production of 6000–15,000 tonnes y^−1^ (estimate obtained combining data from Romero et al.^51^, Mateo and Romero^53^, Khiari et al.^54^, and Telesca et al.^27^) our results give a potential entrapment of 867 million plastic debris in EG each year. How many of these plastic-rich EG are annually flushed ashore is unknown. Given the ever-increasing plastic load reaching our oceans^3^, seagrass ecosystems such as *P. oceanica* meadows will play a crucial role. Therefore, in addition to the key and extensively documented ecosystem services provided by seagrass beds^22,24,55^, *P. oceanica* may provide a valuable added plastic buffering and trapping service. This may be particularly important in the Mediterranean Sea, where this species is endemic, where high microplastics loadings have been found at surface waters and on the seafloor^14,40,43,56^. The declining trend of the areal extent (13–50%), cover (− 1.22% yr^−1^ in average) and shoot density (50% thinning) of *P. oceanica* meadows during the last few decades^27,33^, though de los Santos et al.,^57^ report deceleration trends, points to a severe reduction of the marine plastic trapping role by this seagrass species right when we are starting to realise it.

In addition, besides climate change, spreading of invasive species, excess nutrient inputs, coastal erosion and mechanical impacts^33,58^, plastic pollution may also pose a significant threat to seagrasses around the world. There are some evidence of alteration of seagrass competitive intensity^59^, adherence to seagrass tissues^60^, and consumption by herbivores^61,62^, even though current studies are not sufficient to provide a clear picture of the consequences of plastics in seagrass ecosystems^63^. What is clear is that the deterioration of seagrass meadows may compromise the services they provide, so it is crucial to undertake specific actions to mitigate threats causing regression and ensure conservation.

## Methods

EG and loose leaves were collected in four beaches of Mallorca island in the western Mediterranean Sea: Sa Marina, Son Serra de Marina, Costa dels Pins, in the north-east of the island, and Es Peregons Petits, in the south-west. Beaches were microtidal, gently sloping and composed by medium to fine carbonate-rich sand, and were adjacent to extensive *Posidonia oceanica* meadows. EG and quadrats of loose leaves (500–1000 g) were collected in July–August 2018 and December 2018–January 2019 along 50 m long transects at 0 m, 2.5 m and 7 m of the shoreline.

Once in the laboratory, samples were dried at room temperature (25 °C) and low humidity for several days. We assume low moisture content and comparability of samples (EG and loose leaves) in identical operative conditions. EGs were weighted with an analytical balance with a sensitivity of 0.01 mgr, and the length of their principal axes was measured. EG were then carefully disentangled and fibers sieved at 8 mm, 5 mm, 1 mm and 0.63 mm using stainless steel sieves. Accordingly, the smallest plastic particle size detected was 0.54 mm. The content of the sieves was transferred to petri dishes, and H_2_O_2_ 30% and HCl 10% were added to remove most of the organic matter and calcium carbonate. Samples were oven-dried at 50 °C for > 24 h. The Petri dishes were inspected for plastic debris under a Nikon SMZ1000 stereo-microscope (10 ×–40 ×) coupled with a DS-Fi2 camera in a clean laboratory. All extracted plastic particles were weighted and transferred to a 90 mm Petri dish containing a black and white background that enabled high contrast with plastic colors. The same procedure was applied to loose leaves.

Each Petri dish containing plastic particles was photographed with a Jai 3CCD High Speed Color Line Scan Camera with a 50 mm F2.8–22 lens and a resolution of 150 pixel cm^−1^ at the CORELAB Laboratory of the University of Barcelona using a fixed light temperature (4000 K) and lens aperture (f/11). High-resolution images were processed using the image-processing ImageJ software v1.50i (https://imagej.net/), which allowed gathering information on size (i.e. the maximum distance between any two points calculated at various angles)^40^. Then plastics were classified into four size categories: microplastics (< 5 mm), mesoplastics (5–25 mm) and macroplastics (> 25 mm). Plastic items were also classified according to their nature and shape in fragments, filaments and fibers, films, pellets, sticks and foams. Finally, a subset of 124 particles were randomly selected and chemically identified using a Perkin Elmer Frontier FT-IR Spectrometer with a diamond crystal ATR accessory at the Scientific and Technological Centres of the University of Barcelona (CCiTUB). FT-IR spectroscopy allowed the identification of the polymer composition of each item based on IR absorption bands that represent the presence or absence of specific functional groups in the material. The spectral range analysed was between 4000 and 220 cm^−1^ with a 4 cm^−1^ resolution and 16 accumulations. Each spectrum was compared with known spectrums of Bio-Rad Sadtler Raman Spectra databases (BioRad-KnowItAll Informatics System 2015, Raman ID Expert Inc.).

Samples, sieves and petri dishes were covered wherever possible to minimize periods of exposure. A clean workspace was maintained by keeping all surfaces and equipment clean using ethanol wipes, and cotton clothing was worn.

Pairwise comparisons were performed using the Mann–Whitney U test between unpaired groups. The chi-square test was used to determine the significance between expected and observed frequencies. Furthermore, *t* tests were performed on the regression coefficients to establish statistical significance. All statistical tests was conducted in R (version 3.6.3) using packages dplyr, car, corrplot, qqplotr, gmodels and ggplot2. All values were considered significant when p ≤ 0.05.

## Supplementary Information

Supplementary Information.
