# Supplementary material for: Seagrasses provide a novel ecosystem service by trapping marine plastics
Source: Sci Rep. 2021 Jan 14;11:254. doi: 10.1038/s41598-020-79370-3 (PMC7809288; doi:10.1038/s41598-020-79370-3)
Supplement: Supplementary file 1 — Supplementary Information. [file 41598_2020_79370_MOESM1_ESM.docx]

**Seagrasses provide a novel ecosystem service by trapping marine plastics**

Anna Sanchez-Vidal^1*^, Miquel Canals^1^, William P. de Haan^1^, Javier Romero^2^, Marta Veny^1^

^1^ GRC Geociències Marines, Departament de Dinàmica de la Terra i de l’Oceà, Universitat de Barcelona, 08028 Barcelona, Spain.

^2^ Departament de Biologia Evolutiva, Ecologia i Ciències Ambientals, Universitat de Barcelona, 08028 Barcelona, Spain.

**Supplementary Information**

**Table S1.** Main plastic polymers, density and abundances in aegagropilae (EG) and loose leaves found in beaches.

|  | **Density**  **(g cm^-3^)** | **Abundance**  **in EG** | **Abundance**  **in leaves** |
| --- | --- | --- | --- |
| Polypropylene (PP) | 0.90 | 13.51% | 32.18% |
| Polyethylene (PE) | 0.95 | 21.62% | 50.57% |
| Polybutene (PB) | 0.95 | 2.70% | 0.00% |
| Seawater | 1.02 |  |  |
| Polyestyrene (PS) | 1.06 | 2.70% | 1.15% |
| Polyamide (PA) | 1.16 | 10.81% | 2.30% |
| Polymethyl methacrylate (PMMA) | 1.18 | 0.00% | 1.15% |
| Polyurethane (PU) | 1.20 | 2.70% | 1.15% |
| Polyvinyl chloride (PVC) | 1.3 | 10.81% | 6.90% |
| Polyethylene terephthalate (PET) | 1.37 | 35.14% | 4.60% |

**Table S2.** Abundance of debris per mass unit per season (summer 2018, winter 2018-2019) and area (Sa Marina, Son Serra, Es Peregons, Costa dels Pins) in aegagropilae (EG) and loose leaves.

|  | Summer | Winter | Sa Marina | Son Serra | Es Peregons | Costa dels Pins |
| --- | --- | --- | --- | --- | --- | --- |
| Coordinates |  |  | 39.858 N  3.103 E | 39.737 N  3.224 E | 39.330 N  2.991 E | 39.636 N  3.406 E |
| **Aegagropilae (EG)** |  |  |  |  |  |  |
| Total samples | 66 | 132 | 48 | 44 | 66 | 40 |
| Samples with plastic (%) | 17 (26%) | 17 (13%) | 12 (25%) | 6 (14%) | 15 (23%) | 1 (3%) |
| Average abundance (debris kg^-1^) | 67.16 | 51.62 | 100.07 | 35.35 | 71.43 | 4.31 |
| Maximum abundance (debris kg^-1^) | 581.40 | 1470.59 | 1470.59 | 581.40 | 1020.41 | 172.41 |
| **Loose leaves** |  |  |  |  |  |  |
| Total samples | 21 | 21 | 15 | 9 | 12 | 6 |
| Samples with plastic | 9 (43%) | 12 (57%) | 8 (53%) | 2 (22%) | 7 (58%) | 4 (67%) |
| Average abundance (debris kg^-1^) | 2.56 | 48.48 | 3.44 | 0.50 | 83.36 | 2.60 |
| Maximum abundance (debris kg^-1^) | 13.35 | 613.42 | 13.35 | 2.60 | 613.42 | 10.80 |
